# A practical method for predicting patient‐specific collision in radiotherapy

**DOI:** 10.1002/acm2.12915

**Published:** 2020-05-28

**Authors:** Junjie Miao, Chuanmeng Niu, Zhiqiang Liu, Yuan Tian, Jianrong Dai

**Affiliations:** ^1^ Department of Radiation Oncology National Cancer Center/National Clinical Research Center for Cancer/Cancer Hospital Chinese Academy of Medical Sciences and Peking Union Medical College Beijing China

**Keywords:** collision detection, Pinnacle scripting, radiotherapy, treatment planning

## Abstract

**Purpose:**

To develop a practical method for predicting patient‐specific collision during the treatment planning process.

**Materials and method:**

Based on geometry information of the accelerator gantry and the location of plan isocenter, the collision‐free space region could be determined. In this study, collision‐free space region was simplified as a cylinder. Radius of cylinder was equal to the distance from isocenter to the collimator cover. The collision‐free space was converted and imported into treatment planning system (TPS) in the form of region of interest (ROI) which was named as ROISS. Collision was viewed and evaluated on the fusion images of patient's CT and ROIs in TPS. If any points of patient's body or couch fell beyond the safety space, collision would occur. This method was implemented in the Pinnacle TPS. The impact of safety margin on accuracy was also discussed. Sixty‐five plans of clinical patients were chosen for the clinical validation.

**Results:**

When the angle of couch is zero, the ROISS displays as a series of circles on the cross section of the patient's CT. When the couch angle is not zero, ROISS is a series of ellipses in the transverse view of patient's CT. The ROISS can be generated quickly within five seconds after a single mouse click in TPS. Adding safety margin is an effective measure in preventing collisions from being undetected. Safety margin could increase negative predictive value (NPV) of test cases. Accuracy obtained was 96.3% with the 3 cm safety margin with 100% true positive collision detection.

**Conclusion:**

This study provides a reliable, accurate, and fast collision prediction during the treatment planning process. Potential collisions can be discovered and prevented early before delivering. This method can integrate with the current clinical workflow without any additional required resources, and contribute to improvement in the safety and efficiency of the clinic.

## 1 INTRODUCTION

Collision between the treatment hardware and the patient is a concern in C‐arm linear accelerators. Some cases (i.e., treatment isocenter location, position and immobilization devices) tend to increase the risk of collisions. Such collisions can lead to equipment damage and (or) patient injury during the delivery process. In recent years, radiation treatment planning and delivery techniques have been greatly improved. Noncoplanar treatment plans, which utilize nonzero couch angles, are often used to obtain better dose distributions.^1^, ^2^, ^3^ However, the added geometric complexity increases the risk of collisions between gantry and patient, or gantry and couch.^4^, ^5^ Collision issues are among the most commonly reported incidents for stereotactic body radiation therapy (SBRT)^6^ and the lack of reliable collision avoidance is a critical barrier to the delivery of advanced treatments.^7^, ^8^

Some researchers proposed methods to avoid collisions using supplemental cameras during the delivery process.^9^ And these features of collision avoidance are also available in some linear accelerator devices, such as Varian's LaserGuard collision detection system. Generally, these devices or methods play a very good role in the protection against collisions during the delivery. However those methods do not prevent collision in advance. They can be used as the last line of defense for security. If collisions cannot be avoided during the delivery, re‐planning might be required. Sometimes collisions are not detected until part of the plan has been delivered. This increases the difficulty of re‐planning. An optional solution is to do a collision check before treatment. This collision check in treatment room usually prolongs patient setup and treatment time, which results in a suboptimal clinical flow and may be burdensome for patients.

A number of quality‐control approaches have been developed to mitigate this risk before the delivery process. The most common are gantry/couch angle charts.^10^, ^11^, ^12^ The charts make it efficient for radiation oncology teams to verify whether beam setup will result in collisions between the machine and the patient. But these charts do not take into account of the specific treatment center and additional treatment devices. Some groups have demonstrated collision avoidance using 3D or CAD design systems. In the early days, the location of isocenter was not considered and patient's body was replaced with geometric models.^13^, ^14^ These factors have a great impact on the accuracy of the results. More accurate frameworks have been developed in recent years.^15^, ^16^, ^17^, ^18^ The relevant geometry was modeled from a polygon mesh, and they focused on visualization and moved to a strictly computational solution. Those solutions significantly improved prediction accuracy and could be used in many complex noncoplanar plans. But those software solutions usually require specialized 3D modeling modules, and the construction is complicated. Since those solutions are usually not plugged in the planning system, the application of them remains to be a time‐consuming process.

Although collision detection in radiotherapy has been researched in different forms over the past 10 years, a reliable clinical implementation has not been widely adopted. Our current study aims to provide a simple and accurate patient‐specific collision avoidance method during the treatment planning process. The scripts based on this method which is built in the treatment planning system can be called simply and quickly.

## 2 MATERIALS AND METHODS

### 2.A Model

**FIG. 1:**
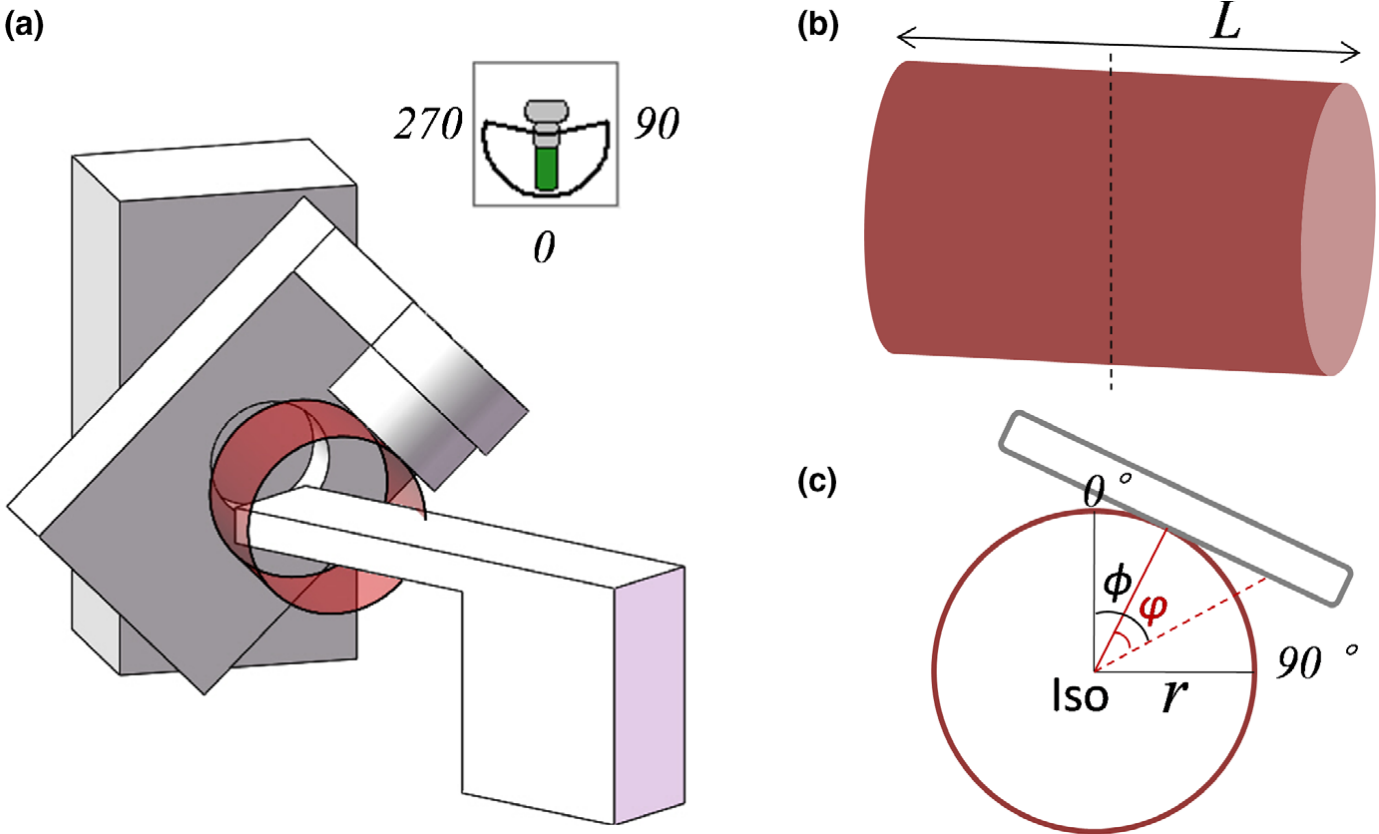
(a) Illustration of collision‐free space of a linac, (b) The cylindrical structure formed by the critical points on the gantry when it rotates along isocenter, where *L* is the length of the treatment head in the superior‐inferior direction, (c) The cross section of the cylinder, where *r* is the radius.

Collision calculations using the methodology presented in this study require information about both the C‐arm linear accelerator and the CT images of patient in the treatment position. The collision‐free space inherent to the linear accelerator is defined by the machine geometric parameters. Previous studies^16^, ^17^ show that each point on the surface of its components describes a circle when the linear accelerator gantry rotates along the isocenter. As shown in Fig. 1, the collision‐free space for the machine is determined by these circles. Although the treatment head has pieces that protrude farther than others, it is simplified here as a flat surface, so the radius of the clearance circle remains unchanged along the longitudinal (superior‐inferior) direction. Patient's body and couch beyond these circles are at risk for collision. For collisions are usually determined by the smallest circle, the radius of smallest circle could be chose as the radius of the whole structure for safety and simplicity. In the “rooms‐eye view”, the collision‐free space is a cylinder with radius equal to the distance between the isocenter and front cover of collimator. The height of cylinder is defined by the length of the treatment head in the superior‐inferior direction. The radius can serve as a threshold for collision detection, and the height can be used to determine the longitudinal range of the patient.

**FIG. 2:**
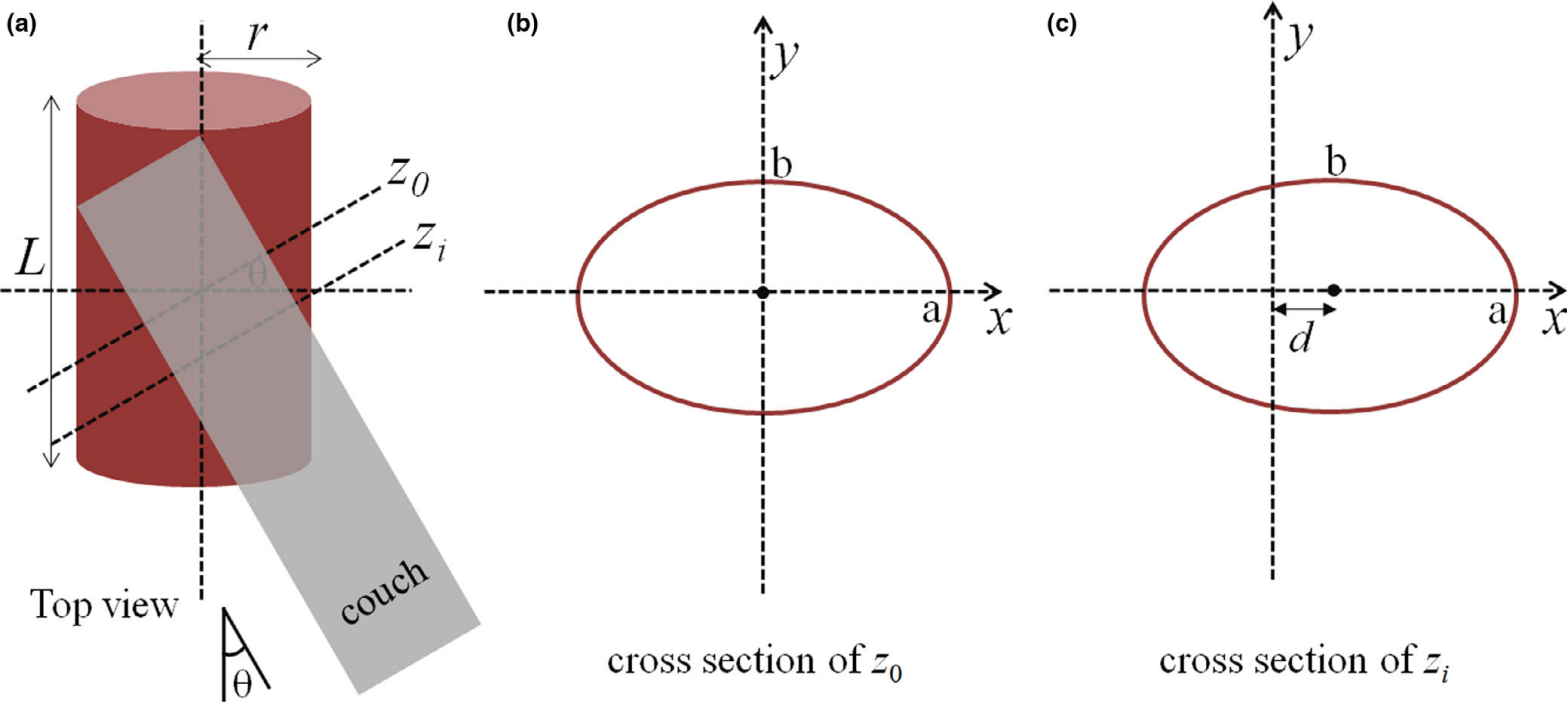
(a) Illustration of collision detection with a couch angle *θ* in top view, (b) the cross section passing through the treatment center (*z_0_*), (c) a cross section for nonisocenter (*z_i_*).

When isocenter of the patient's treatment plan is selected, the relative position between the collision‐free space and the patient's CT is determined. The structure of collision‐free space can be fused to the patient's CT images by transformation. In this paper, the structure of collision‐free space was imported into the TPS in the form of region of interest (ROI). ROI is used in almost all planning systems and can be visualized in various displays. Thus, this method can be highly integrated with existing commercial TPS. The treatment planner can see clearly the relative position between the patient body (or couch) and the ROI of safe space (ROISS) in the planning system. The shape and position of ROISS on patient's CT images is determined by plan isocenter and machine parameters. When the couch angle is *θ*, the shape of ROISS is an oval on patient's cross section. As shown in Fig. 2, the elliptic equation on the cross section of treatment center (*z_0_*) is:

(1) $$\frac{x^{2}}{a^{2}}+\frac{y^{2}}{b^{2}}=1,$$

where *a* = *r*/cos*θ*, *b* = *r*, *r* is the radius of cylindrical structure. The elliptic equation on the cross section of nontreatment center (*z_i_*) is:

(2) $$\frac{{\left(x-d\right)}^{2}}{a^{2}}+\frac{y^{2}}{b^{2}}=1,$$

where *d*=|*z*1‐*z*0|·tan *θ*. From Eqs. (1) and (2), it is clear that the shape of ROISS will be a series of same circles on CT images when the couch angle is zero (coplanar plan).

### 2.B Clinical implementation

The technique presented in this paper was implemented in the Pinnacle^3^ 9.10 (Philips Radiation Oncology Systems) treatment planning system. Varian Edge accelerator with Qfix couch was used in this study, and the size of couch and gantry have been measured. The patients were scanned with a Phillips Brilliance Big Bore CT using 3 mm slice thickness, and the images were sent to Pinnacle TPS. ROI of couch structure was generated in place of CT couch which might lack detail and accuracy. ROI of couch was created by Pinnacle Scripts automatically. Contours of patient were contoured automatically using a density threshold of 0.6 g/cm^3^. The isocenter coordinates, ROI contours, couch angle, image coordinates, and beam information were read from the TPS by Pinnacle Scripts. Then this plan information together with treatment head geometry was sent to the program written in python (version 2.7.10).

**FIG. 3:**
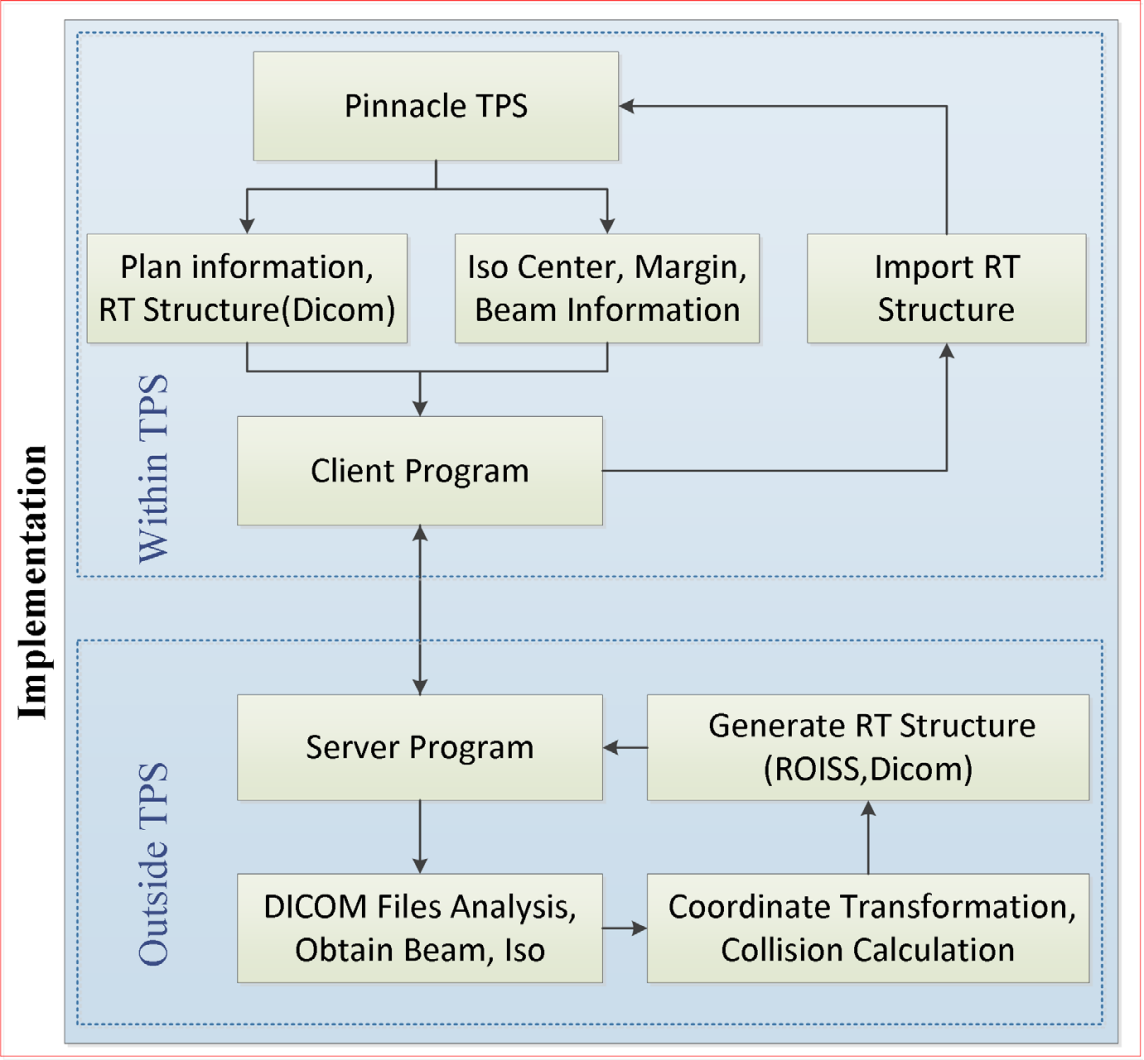
Flowchart of the implementation process.

In the python program, the isocenter coordinates were used as a point of origin. The safe space was generated. Then the space was converted into ROISS. The ROISS structure was written to DICOM file or Pinnacle ROI file. At last, ROISS structure was imported into TPS by Pinnacle Scripts. If the couch angles of all the plan fields were the same, only one ROISS would be created. For the plans with multiple couch angles, a ROISS would be created for each beam field. The order and name of ROISS were set the same as those of the beam field to eliminate possible confusion. A flowchart depicting this process is shown in Fig. 3.

### 2.C Collision detection

Collision was viewed and evaluated on the fusion images of patient's CT and ROIs in TPS. If any points of body contour or couch fell beyond the ROISS structure, collision would occur. This process could be implemented easily by ROIs subtract function provided by TPS. If the volume of either the couch or the body contour beyond the ROISS was zero, a possible collision would happen.

When a possible collision is detected, the program will further calculate the gantry angle range that would result in collision. Based on the published study,^16^ the angle range of a collision is $\left(\phi^{\text{r max}}-\varphi \right)\to \left(\phi^{\text{r max}}+\varphi \right)$ , where *ϕ* ^r max^ is the angle value (polar coordinates) of the patient (or couch) point of maximum collision in a certain region. The variable *φ* is determined by equation

(3) $$\varphi =\arccos\frac{r}{\sqrt{{\left(x^{r\max}\right)}^{2}+{\left(y^{r\max}\right)}^{2}}},$$

where *x^r^* ^max^ and *y^r^* ^max^ are the coordinate value (Cartesian coordinates) of the point of maximum collision in a certain region, *r* is the radius of cylindrical structure as shown in Fig. 1(c). Rotation transformation of coordinates is required when using noncoplanar plan fields. The new coordinates of the ROI points follow

(4) $$\vec{V^{\prime }}=R_{\mathrm{couch}}\cdot \;\vec{V},$$

where the transformation matrix is

(5) $$R_{\mathrm{couch}}=\left[\begin{array}{ccc} \cos\theta & \sin\theta & 0\\ -\sin\theta & \cos\theta & 0\\ 0 & 0 & 1 \end{array}\right],$$

*θ* is the couch angle, $\vec{V}={\left[x\;y\;z\right]}^{\prime }$ is the coordinate vector of ROI point. Then the angle range of the collision is recorded for the corresponding ROISS. If the angle range of the collision overlaps with the angle range of corresponding plan fields, the TPS script will register the event as a collision and warn the user that the plan is unsafe to deliver. For easy identification, the ROISS is marked green if there is no collision, and the ROISS is marked red if there is a collision.

A safety margin can also be added to the calculation to account for factors such as possible patient position variations, uncertainties in the input data, or error of geometric measurement. This margin is determined by the user. The model applies it for calculation by reducing the clearance radius.

### 2.D Clinical validation

Sixty‐five plans of patients from May 2018 to October 2018 at our institution were randomly selected for the clinical validation, including brain, lung, rectum, breast, liver, and osteosarcoma cases. Each plan was designed with 1–4 arcs. The total number of arc fields was 162 and the corresponding couch angle (beam number) of fields included 0°(65), 20°(34), 30°(32), 45°(31). The protocol for this study was approved by our institutional review board and each patient consented to the study prior to enrollment. For collision prediction techniques, it entails comparing the calculated collision and measured physical collision. Collision prediction accuracy was assessed for various safety margins using receiver operating characteristic (ROC) curve analysis as recommended by Cardan et al.^17^ Definitions for the ROC categories are shown in Table 1.

**TABLE 1:** Definitions of categories for ROC analysis with collision detection.

Category	Calculated collision	Measured collision
True positive (TP)	Yes	Yes
True negative (TN)	No	No
False positive (FP)	Yes	No
False negative (FN)	No	Yes

## 3 RESULTS

### 3.A Clinical implementation

**FIG. 4:**
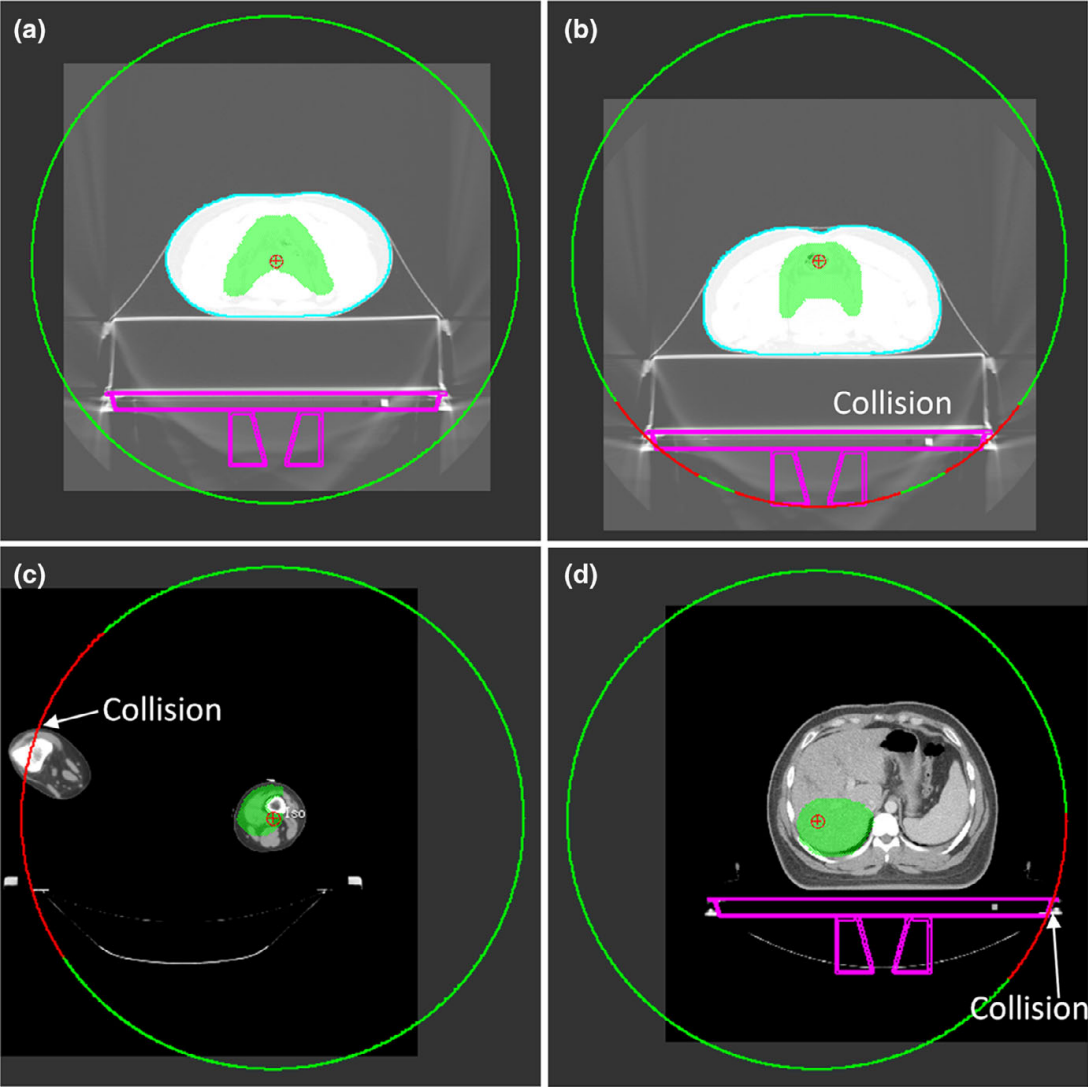
(a) A safe rectal plan, (b) a rectal plan in which gantry‐couch collision would happen, (c) a plan for leg position in which gantry would collide with patient, (d) a liver plan in which gantry would collide with couch. The couch angle in these plans is 0°. The range where collision occurs is colored in red, and the remaining regions are in green.

When the couch angle is zero (coplanar plan), ROISS generated in the Pinnacle is shown in Fig. 4. The ROISS is a cylindrical structure which contains a series of identical circles. Four examples based on a full gantry rotation are shown in Fig. 4. Figure 4(a) shows a safe rectal plan in which the patient and couch are within the safe space. In Fig. 4(b), gantry‐couch collision will happen in a rectal plan because the thickness of the prone plate is large and the location of treatment center is too high. Similar collisions can also occur in the Fig. 4(d) in a liver plan with treatment center on the right side of the patient. A gantry‐patient collision can be predicted for a leg position plan when the patient is fixed with a specific posture as shown in Fig. 4(c).

**FIG. 5:**
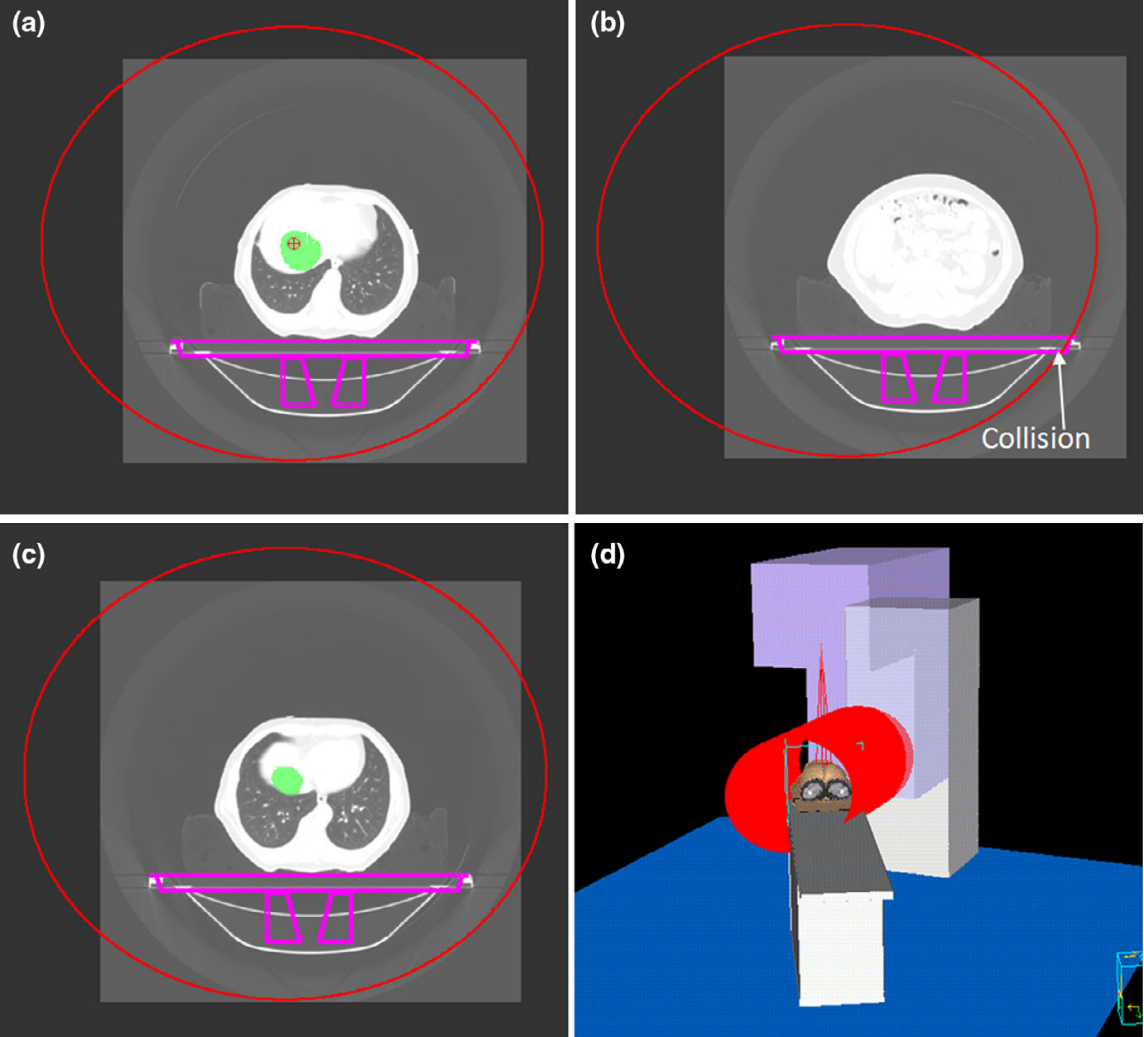
For a liver plan in Pinnacle TPS, ROISS structure is shown as a series of ovals on CT images. (a) is the cross section passing through treatment center, (b) and (c) are the cross sections with off‐center ovals for nonisocenter, (d) shows the three dimensional structure of trajectory ROI in the rooms‐eye view within Pinnacle TPS. The couch angle in this plan is 30°.

When noncoplanar plan is designed (the couch angle is not zero), ROISS is an oval structure in the transverse view of patient CT. Figure 5 shows the ROISS structure with different cross sections for a liver plan in Pinnacle TPS when the couch angle is 30°. Figure 5(a) is the cross section passing through treatment center. Figure 5(b) and 5(c) are the cross sections with off‐center ellipses for nonisocenter level, and the offset distance in the *x* direction is determined by *d* in Eq. (2). Figure 5(d) shows the three dimensional structure of ROISS in the rooms‐eye view within Pinnacle TPS.

### 3.B Clinical validation

**FIG. 6:**
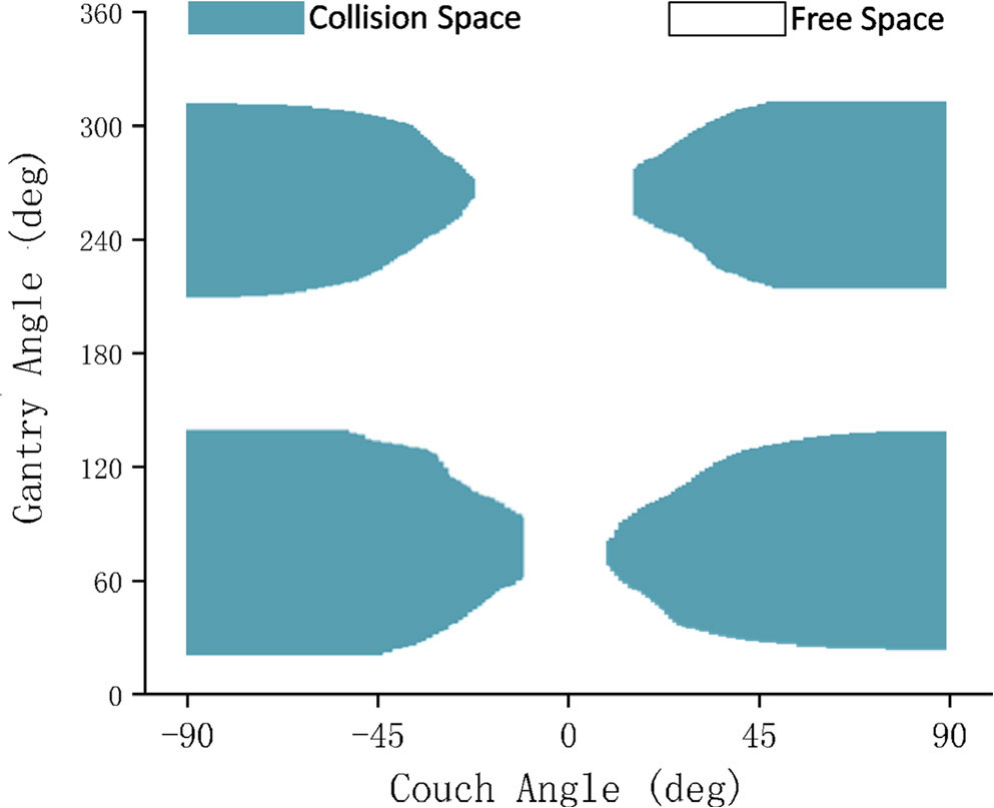
Collision map of gantry angle vs couch angle for an abdominal case. −90° on the x‐axis corresponds to 270° in the IEC 61217 coordinate system shown in Fig. 1(a).

**FIG. 7:**
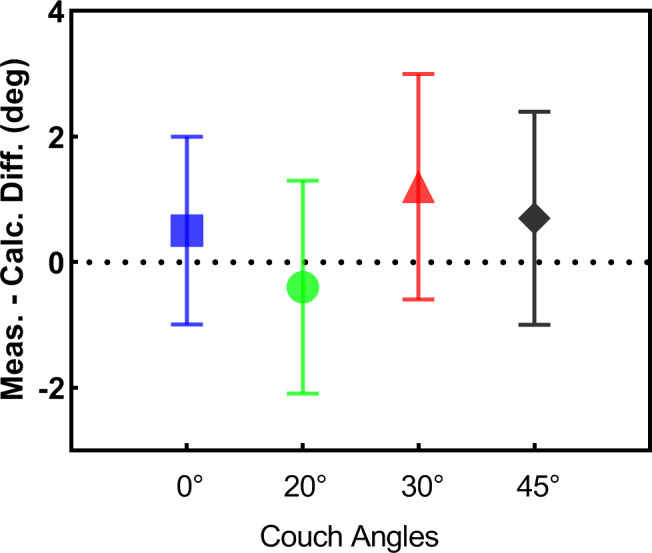
The difference between the measured and calculated collision angle with different couch angles.

Figure 6 shows the delineation of the calculated collision space and free space for an abdominal case. The average difference between measured and calculated collision angle was 0.7°with a standard deviation of 1.6°. The detailed statistics for the selected couch angles could be seen in Fig. 7. It can be easily deduced from this figure that the accuracy has little relation with the selected couch angle.

Table 2 shows ROC results with varying safety margins for the test cases. It is clear that adding a safety margin increases the negative predictive value (NPV) for the tests. The increase in NPV results in more false positives which lower the overall accuracy. Accuracy drops from 97.5% to 94.4% between the 0 and 5 cm safety margins. Larger safety margins reduce the number of false negative results with a subsequent increase in false positives. Appropriate margin choice is a trade‐off between preventing undetected collisions and reduction in accuracy.

**TABLE 2:** ROC results for the test cases with three different safety margins. Accuracy is given by the sum of true positive (TP) and true negative (TN) results divided by the total for all results. Negative predictive value (NPV) = TN/(TN + FN).

	TP|TN|FP|FN	Accuracy (%)	NPV (%)
No safety margin	62|96|1|3	97.5	97.0
1 cm safety margin	65|92|3|2	96.9	97.9
3 cm safety margin	69|87|6|0	96.3	100.0
5 cm safety margin	71|82|9|0	94.4	100.0

This method with 3 cm safety margin has been used in our hospital for more than 800 cases since October 2018. The collision in radiotherapy has been nearly eliminated in our clinic. It will take about five seconds after click of the mouse to generate ROISS structure in the planning process. For this method can be highly integrated with TPS, it is convenient and simple for clinical use.

## 4 DISCUSSION

In this study, we have established a new method to implement patient specific collision avoidance during the treatment planning process. ROISS was achieved by converting the collision‐free space region into an ROI structure in TPS. Collision could be viewed and evaluated visually on the fusion images of patient's CT and ROIs in TPS. This process was successfully implemented in the pinnacle planning system through scripting and python code.

Through ROC analysis, a 3.0 cm safety margin could increase NPV of test cases to 100%. A safety margin with 3.0 cm or more is shown to be sufficient in preventing test case collisions from being undetected. As shown in Table 2, the clinically used margin of 3 cm reduces the accuracy from 97.5% to 96.3%. However, for a clinical setting, it is highly preferable that the safety margin is over 3 cm. If the safety margin is less than 3 cm, it will usually trigger the security alert (e.g., Varian's LaserGuard) when delivering the treatment plan. So a 3–5 cm safety margin is highly recommended for this method.

Several collision prediction methods have been studied. For example, collision indicator charts for gantry‐couch angle combinations for Elekta, Varian, and Siemens machines have been published.^10^, ^11^, ^12^ These charts are quite useful as a quick reference to get a general idea of the collision for a common plan, but might yield misleading results if heavily relied upon for patient‐specific collision assessments. Some computer code or CAD design that modeled the machine and patient for collision detection was also created. However, the patient was represented by a cylinder in some study.^14^ The simplified patient model can have a great effect on the accuracy of the results. Some researchers built the relevant geometric model using polygon mesh,^17^, ^18^ and they detected the collision using complex algorithms. These software solutions usually require specialized 3D modeling modules, and the construction is complicated. As these algorithms are usually not built in the planning system, a time‐consuming and messy process is needed for treatment planners.

For clinical convenience, efficient and highly integrated collision prediction methods within TPS are needed. Scripting^19^ and tool (eg ClearCheck) integrated with Eclipse TPS have been developed. In this study, we provide a more practical and general method which can be highly integrated with multiple TPSs. Since intuitive judgment can be made in the planning system, this method can be used not only for collision detection, but also for selecting the appropriate treatment center in planning design.

There are situations when a collision occurs outside the anatomy included in the planning CT. This issue may limit the accuracy of collision prediction based on CT scan. Some studies^7^, ^15^, ^16^ used a 3D scanning technique to acquire relatively accurate body contours. The 3D scanning technique approaches can improve prediction accuracy, but at the expense of additional equipment cost and scanning time. Padilla et al. estimated that a total time of 10 min was needed for an experienced user. Since the 3D scanning process is usually implemented during treatment simulation, it results in a suboptimal clinical flow and may be burdensome for some patients. Compared with collision prediction based on 3D scanning technique, CT simulation‐based techniques are limited by the scan range for some patients but require little additional clinical resources. Collision prediction using CT‐scanned anatomy is usually implemented during planning design and does not take up additional time of patient. To eliminate potential limit caused by missing CT images, one method is to artificially extend the CT images to replace the missing images. The collision detection in this case may be inaccurate when there is a big difference between the shape of the added body contours and actual ones. Another possible method is to predict missing images based on existing CT images and deep learning method. This method may be implemented in future research.

Compared with previous published works^17^, ^19^, the detection accuracy is similar. In our clinical validation, there are four patients with failed predictions. Three patients were treated for lung tumors and were simulated with arms‐up. The CT images did not include the scan of the full arms. The main reason was that the real missing images of arms were different from the contours what we added. One patient was treated for osteosarcoma which was located in the left leg. The gantry collided with the right leg which was out of the range of plan field. The reason for this was because treatment position of the healthy leg was not strictly repeated according to simulated position. This problem can be easily solved by strict position for all body parts of the patient.

In this study, we established a method to convert the collision‐free space region into an ROI structure in TPS. The method was implemented with Pinnacle Scripting and python code. This method can be highly integrated with TPS and clinical workflow. The processes of exporting, processing, and importing patient plan data was finished with code automatically. Collision can be viewed and evaluated on the fusion images of patient's CT and ROIs in TPS automatically. The collision detection method has been purposefully designed to be easily usable at our clinic. Although this method was implemented using a Varian EDGE linac, it could be applied to other types of accelerators after a simple modification of the input parameters. Also this method could be used in other TPS with proper corresponding code.

It should be noted that the collision‐free space region of gantry is simplified as cylindrical area in this method. Any further protrusions of the collimator can be modeled as separate clearance cylinders, if desired. For collisions are usually determined by the smallest circle, the radius of smallest circle is chose as the radius of the whole structure for safety and simplicity. Although this simplicity guarantees safety of treatment plan, it will affect the accuracy of the prediction. For more accurate predictions, more elaborate models could be considered in the future.

## 5 CONCLUSIONS

This paper has demonstrated the feasibility of a new method for collision detection. It provides a reliable, accurate, and fast collision prediction during the treatment planning process which allows revision of the treatment plan. The use of TPS Scripting enables maximal integration with the current clinical workflow without any additional required resources. Potential collisions can be discovered and prevented at the stage of treatment planning process. If included in the clinical flow, this method can minimize re‐planning due to collisions, and contribute to improvement in the safety and efficiency of the clinic.

## CONFLICTS OF INTEREST

The authors declare no conflict of interests.
